# PD43 Single-Arm Studies In Literature Reviews: Trials Versus Case Series

**DOI:** 10.1017/S0266462324003064

**Published:** 2025-01-07

**Authors:** Mary Chappell, Anita Fitzgerald, Alice Sanderson, Deborah Watkins, Paul Miller, Lavinia Ferrante di Ruffano, Mary Edwards, Rachael McCool

## Abstract

**Introduction:**

Single-arm studies, particularly single-arm trials (SATs), are increasingly being used in submissions for marketing authorization and health technology assessment. As reviewers of evidence, we sought to better understand the validity of SATs, compared with observational single-arm studies (case series), and how to assess them in our reviews.

**Methods:**

We conducted a highly pragmatic literature review to create a convenience sample of recent systematic reviews published from January to July 2023 to establish the following: (i) what single-arm study designs are included; (ii) what quality assessment tools are used; and (iii) whether there is a difference in effect size and variability among different study designs. A single reviewer identified reviews of interventions that included single-arm studies and extracted information on the numbers of included SATs and case series, and the quality assessment tools used. Any misclassifications by review authors were identified. For meta-analyses, outcome data were extracted and a subgroup analysis comparing SATs and case series was conducted.

**Results:**

Work is still underway to complete this investigation. So far, it appears that a large proportion of systematic reviews misclassify SATs and case series studies and few use appropriate quality assessment tools. There is not yet any evidence of a systematic difference between SATs and case series in terms of effect size.

**Conclusions:**

Findings suggest that there is poor understanding of SATs in the review community. There are limited specific quality assessment tools for SATs and review authors frequently use inappropriate tools to assess them. More research is likely to be needed to investigate the relative validity of SATs and single-arm observational studies.

